# Retraction: MiR-22 inhibition alleviates cardiac dysfunction in doxorubicin-induced cardiomyopathy by targeting the sirt1/PGC-1α pathway

**DOI:** 10.3389/fphys.2022.1020568

**Published:** 2022-08-24

**Authors:** Frontiers Editorial Office

Following publication, concerns were raised regarding the integrity of the images in the published figures. The authors failed to provide a satisfactory explanation during the investigation, which was conducted in accordance with Frontiers’ policies.

Given the concerns, the editors no longer have confidence in the findings presented in the article.

During correspondence, the authors did not respond to the question of whether they agree or disagree to the retraction.

This retraction was approved by the Chief Editors of Frontiers in Physiology and the Chief Executive Editor of Frontiers.

